# Comparison of ten policy options to equitably reduce children’s exposure to unhealthy food marketing

**DOI:** 10.1017/S1368980024000958

**Published:** 2024-04-29

**Authors:** Ryan Gage, Wei Liu, Amber L Pearson, Moira Smith, Michelle Barr, Ashton Shortridge, Louise Signal

**Affiliations:** 1 Department of Public Health, University of Otago, Wellington, 6242, New Zealand; 2 Department of Geography, Environment and Spatial Sciences, Michigan State University, East Lansing, MI 48824, USA; 3 Department of Water Ecology and Environment, China Institute of Water Resources and Hydropower Research, Beijing 100038, China; 4 Charles Stewart Mott Department of Public Health, Michigan State University, Flint, MI 48502, USA

**Keywords:** Food marketing, Food advertising, Health policy, Children, Obesity

## Abstract

**Objective:**

Reducing children’s exposure to unhealthy food marketing is crucial to combat childhood obesity. We aimed to estimate the reduction of children’s exposure to food marketing under different policy scenarios and assess exposure differences by socio-economic status.

**Design:**

Data on children’s exposure to unhealthy food marketing were compiled from a previous cross-sectional study in which children (*n* 168) wore wearable cameras and Global Positioning System (GPS) units for 4 consecutive days. For each exposure, we identified the setting, the marketing medium and food/beverage product category. We analysed the percentage reduction in food marketing exposure for ten policy scenarios and by socio-economic deprivation: (1) no product packaging, (2) no merchandise marketing, (3) no sugary drink marketing, (4) no confectionary marketing in schools, (5) no sugary drink marketing in schools, (6) no marketing in public spaces, (7) no marketing within 400 m of schools, (8) no marketing within 400 m of recreation venues, (9) no marketing within 400 m of bus stops and (10) no marketing within 400 m of major roads.

**Setting:**

Wellington region of New Zealand.

**Participants:**

168 children aged 11–14 years.

**Results:**

Exposure to food marketing varied by setting, marketing medium and product category. Among the ten policy scenarios, the largest reductions were for plain packaging (60·3 %), no sugary drink marketing (28·8 %) and no marketing in public spaces (22·2 %). There were no differences by socio-economic deprivation.

**Conclusions:**

The results suggest that plain packaging would result in the greatest decrease in children’s exposure to food marketing. However, given that children are regularly exposed to unhealthy food marketing in multiple settings through a range of marketing mediums, comprehensive bans are needed to protect children’s health.

## Introduction

Restricting unhealthy food marketing (hereafter food marketing) is a recommended action to address high and inequitable rates of childhood obesity^(1,2)^. Children are exposed to around twenty-seven unhealthy food marketing advertisements a day, more than twice that of healthy food marketing, according to a New Zealand study, Kids’Cam, conducted by members of this research team in 2014/15^(3,4)^. Exposure to food marketing has been associated with increased preferences, attitudes and consumption of the marketed products^(5)^. Moreover, there is evidence that children are particularly susceptible to marketing and may change behaviours after brief bouts of exposure^(6)^.

There is a growing consensus that exposure to unhealthy food marketing is a breach of children’s rights under the United Nations Convention on the Rights of the Child^(7)^. Under the Convention, children have the right to protection from commercial activities that infringe on children’s rights, including food marketing. Recently, governments have been challenged to ‘regulate targeted or age-inappropriate advertising, marketing and other relevant digital services to prevent children’s exposure to the promotion of unhealthy products, including certain food and beverages’ ^((8), p.16)^.

The WHO recommends that the marketing of foods high in fat, sugar and salt should be prohibited in settings where children gather^(9)^. Restrictions have been placed on marketing in public spaces such as around schools^(10)^, public transport networks^(11)^ and within schools^(12)^. Moreover, bans have been introduced on particular categories of food in particular places^(13)^, e.g. foods high in fat, sugar and salt^(14)^ and on energy drinks in schools and public buildings in Latvia^(12)^. Furthermore, regulations have targeted specific marketing mediums, e.g. television. While restrictions on food packaging are uncommon^(13)^, such regulation has been identified as important, given that bright colours and the usage of cartoons and characters on packaging attract attention and ultimately consumption^(5,15)^.

Little is known about the relative effectiveness and socio-economic equity impacts of different food marketing restrictions on children’s exposure to food marketing. Moreover, research that has been done has relied on self-report or reports of parents/caregivers, which is subject to bias and reporting errors^(16)^.Thus, objective evidence on children’s exposure to food marketing is needed. Additionally, identifying equitable approaches to restrict food marketing is critical for setting priorities and informing policy, particularly because a recent review found children’s exposure to unhealthy food advertising varies by ethnicity and socio-economic status^(17)^.

In 2019, our team combined objective measures of food marketing (captured with wearable cameras) with geospatial methods to estimate the effectiveness of various marketing restriction scenarios in Wellington City^(18)^. The research identified several effective scenarios to reduce children’s exposure to food marketing outdoors, with bans within 400 m of schools, residential areas and playgrounds reducing children’s exposure by 25 %, 27 % and 33 %, respectively^(18)^. However, the study’s inclusion of a single city in a metropolitan region limited the generalisability of the findings. Likewise, only marketing in public spaces was explored, excluding product packaging and exposures at home and school. Further, differences by socio-economic deprivation were not explored.

In this study, we expand on this work by calculating a broader range of policy scenarios across four adjacent cities, incorporating a wide range of marketing mediums, product categories and settings and by socio-economic deprivation. We aimed to estimate the reduction of children’s exposure to food marketing under different policy scenarios and assess differences by socio-economic status.

## Methods

### Study area

The study area is the Wellington region located at the southern end of the North Island of New Zealand, including four adjacent cities: Wellington (290 km^2^), Porirua (182 km^2^), Lower Hutt (377 km^2^) and Upper Hutt (540 km^2^). Wellington city is the region’s most populated city (population = 215 400 in 2019), followed by Lower Hutt (104 900), Porirua (55 500) and Upper Hutt (41 000)^(19)^. To the best of our knowledge, none of these cities have regulations to protect children from unhealthy food marketing and nationally there are only restrictions on broadcast media.

### Study sample

The study sample included children who took part in the Kids’Cam wearable camera study. Kids’Cam was a cross-sectional observational study of 168 randomly selected children (11–14 years) from sixteen randomly selected schools in the Wellington region of New Zealand. Children were selected using a stratified random sampling method, described elsewhere, to provide equal explanatory power by ethnicity and deprivation^(3,4)^. Each child was provided with a wearable camera and GPS device, which they wore on lanyards around their necks. The cameras passively captured a 136-degree image every 7 s, while the GPS devices recorded latitude and longitude every 5 s. Children wore the devices for four consecutive days (t2 weekdays plus 2 weekend days), capturing a total of 1·3 million images and 3 million coordinates. Data were collected between July 2014 and June 2015.

### Coding of unhealthy food marketing

Data on children’s exposure to food marketing were obtained from an existing analysis of the Kids’Cam data^(3)^. Marketing was defined as ‘any form of commercial communication or message that is designed to, or has the effect of, increasing recognition, appeal and/or consumption of particular products and services’^((20), p.9)^. Five experienced researchers coded the presence of marketing, including the setting of exposure (home, school, food venues, recreation venues and other public spaces such as other retail, shop fronts and streets), marketing medium and product category. Before commencing, each coder achieved 90 % agreement on a test set of images. To avoid overestimation or misclassification of exposure, children were considered as exposed to marketing if 50 % or more of the marketing content (e.g. brand, logo and slogan) was in clear view.

A marketing exposure was defined as ‘starting on the first instance of an image with a particular setting/medium/product code; subsequent images were counted as part of the same exposure. An exposure was considered to have ended when 30 s had elapsed since the last recorded code of that setting/medium/product code (defined using the image timestamps). Any subsequent code for that same combination after this time limit was counted as the start of a new exposure sequence’^((3), p.3)^ (see^(3)^ for further details). Food marketing was classified as unhealthy using the WHO Nutrient Profile Model^(21)^ and included the following food/beverage categories: sugary drinks, fast food, confectionary, snack foods, ice cream, diet soft drinks, cookies/cakes/pastries, unhealthy milk products (> 15 g/100 g total sugars) and unhealthy cereals (> 10 g/100 g total sugars).

### Policy scenarios to reduce children’s exposure to unhealthy food marketing

We selected ten core policies for our analysis based on WHO recommendations^(1)^, the existing analysis and the literature (see Table 1). These policies aim to eliminate exposure to multiple forms of marketing by targeting specific settings of exposure, marketing mediums and/or product categories. For example, the first scenario, plain packaging, targets all exposures on food packages/containers, while the second scenario, no marketing of sugary drinks, targets a single food/beverage product. Table 1 summarises the definition of each policy scenario and the food marketing exposures it would avert.

**Table 1:** Policy scenario definitions

Policy	Description	Marketing exposures avoided
1. No marketing on product packaging	Eliminating unhealthy food marketing on food/beverage packages/containers.	Unhealthy food marketing on product packaging (all settings).
2. No marketing on merchandise	Eliminating unhealthy food marketing on product merchandise, defined as branded products used to promote a food or beverage product, e.g. t-shirts, caps and drink bottles.	Unhealthy food marketing on merchandise (all settings).
3. No marketing of sugary drinks	Eliminating marketing of sugary drinks and juices, including flavoured milks. Does not include artificially sweetened beverages.	Marketing of sugary drinks and juices (all settings and marketing mediums).
4. No marketing of confectionary in schools	Eliminating the marketing of confectionary in schools.	Marketing of confectionary in schools (all marketing mediums).
5. No marketing of sugary drinks in schools	Eliminating the marketing of sugary drinks and juices in schools, including flavoured milk. Does not include artificially sweetened beverages.	Marketing of sugary drinks and juices in schools (all marketing mediums).
6. No outdoor marketing in public spaces	Eliminating the display of food marketing in public spaces, excluding product packaging, e.g. on signs, shopfronts, vending machine exteriors. Public spaces include streets, community venues, fresh food markets, sport settings, outdoor recreation spaces and public transport facilities.	Unhealthy food marketing in public spaces, excluding product packaging, e.g. on signs, shopfronts, vending machine exteriors.
7. No outdoor marketing in public spaces within 400 m of schools	Eliminating marketing in public spaces within 400 m of schools, excluding product packaging. Public spaces include streets, community venues, fresh food markets, sport settings, outdoor recreation spaces and public transport facilities.	Unhealthy food marketing in public spaces within 400 m of schools, excluding product packaging, e.g. on signs, shopfronts, vending machine exteriors.
8. No outdoor marketing within 400 m of recreation venues	Eliminating outdoor marketing within 400 m of recreational facilities, excluding product packaging. Recreation facilities include swimming pools and fitness centres.	Unhealthy food marketing in public spaces within 400 m of recreation spaces, excluding product packaging, e.g. on signs, shopfronts, vending machine exteriors.
9. No outdoor marketing within 400 m of bus stops	Eliminating outdoor marketing within 400 m of bus stops, excluding product packaging.	Unhealthy food marketing in public spaces within 400 m of bus stops, excluding product packaging, e.g. on signs, shopfronts, vending machine exteriors.
10. No outdoor marketing within 400 m of main roads	Eliminating outdoor marketing within 400 m of main roads (three or more lanes), excluding product packaging.	Unhealthy food marketing in public spaces within 400 m of main roads, excluding product packaging, e.g. on signs, shopfronts, vending machine exteriors.

### Statistical analyses

Analyses were done in three stages. First, we identified children’s number of exposures to food marketing within each setting, marketing medium and product category of interest. We then identified the number of exposures that would be reduced under each scenario. For policies restricting unhealthy food marketing around schools, recreational facilities, bus stops and main roads (policies 7–10 in Table 1), exposure to unhealthy food marketing in outdoor public spaces was calculated using published methods^(22)^. The best spatial data available for generating these restricted zones were obtained from open online sources including Data.govt.nz (www.data.govt.nz), Land Information New Zealand data (data.linz.govt.nz), Ministry for the Environment Data Service (data.mfe.govt.nz) and the Greater Wellington Regional Council Open Data Portal (data-gwrc.opendata.arcgis.com). 2008 data were available for schools and major roads, 2018 data for recreational facilities and 2019 data for bus stops. While these data were not collected at the same time as the image data, they characterise facilities that are unlikely to have changed over this time.

Second, we calculated the exposure reduction (expressed as a percentage) by dividing the number of food marketing exposures that would be avoided in each scenario by children’s total exposure to food marketing, multiplied by 100. The denominator for this calculation was children’s total exposure to food marketing across all settings, marketing mediums and product categories. For simplicity, we rescaled children’s marketing exposure to daily exposure rates, where one day is equivalent to 10 recording hours. While this does not affect the exposure reduction, it provides a clearer indication of the absolute number of marketing exposures that could be avoided under each scenario. Exposure rates were calculated using Poisson regression.

Third, to compare differences in policy effectiveness by socio-economic deprivation, we created two groups based on the individual deprivation of the child’s caregiver (NZiDep)^(23)^. An individual’s NZiDep score is based on a series of questions that relate to eight deprivation characteristics (e.g. ‘using food grants/banks’, ‘feeling cold to save heating costs’ and ‘out of work for more than one month’^(23)^). NZiDep is a five-point index, with index scores ranging from one (no deprivation characteristics reported) to five (five or more deprivation characteristics reported). NZiDep scores were grouped into low (score 1,2) and high (scores 3,4,5) deprivation categories.

We used R 3.5.3^(24)^ to link food exposures with GPS data and STATA/SE 17 to collate food marketing exposures and analyse policy effectiveness. Results were weighted for the stratified sampling design using STATA’s svy weights, which adjusted for the differential selection of schools by ethnicity and school tertile appropriate to the sampling method applied.

## Results

### Sample characteristics and unhealthy food marketing exposures

Children from low socio-economic deprivation households comprised 50·6 % of the sample, and children from high socio-economic deprivation households comprised 45·8 % (Table 2). Gender and age distributions were similar between deprivation strata, but the high deprivation group had more Māori and Pacific children and fewer NZ Europeans, reflecting deprivation by ethnicity in this colonial country.

**Table 2:** Sample characteristics. Values are numbers (%)

		All children (*n* 168)		Low socio-economic deprivation (*n* 85)*		High socio-economic deprivation (*n* 77)*	
Variable, *n* (%)	Group	*n*	%	*n*	%	*n*	%
Gender	Female	89	53·0	45	52·9	42	54·5
	Male	79	47·0	40	47·1	35	45·5
Age (years)	11	13	7·7	6	7·1	7	9·1
	12	122	72·6	66	77·6	56	72·7
	13	26	15·5	12	14·1	13	16·9
	14	1	0·6	0	0·0	1	1·3
Ethnicity	NZ European	66	39·3	39	45·9	26	33·8
	Māori	60	35·7	25	29·4	30	39·0
	Pacific	42	25·0	21	24·7	21	27·3

* Age missing for six participants; household socio-economic deprivation missing for six participants.

Table 3 summarises children’s mean rate of exposure to unhealthy food marketing by setting, marketing medium and product category for all children and by socio-economic deprivation. On average, children were exposed to 31·7 instances of unhealthy food marketing per day. The highest rate of exposure to unhealthy food marketing occurred in other public spaces (34·0 % of total), followed by at home (28·5 %, Table 3). Most exposures involve product packaging (55·6 %), followed by signage (32·0 %). Sugary drinks were the most common type of unhealthy food marketing exposure (28·6 %), followed by fast food (18·7 %) and confectionary (9·4 %). Exposure settings, marketing medium and product categories for unhealthy food marketing exposures were similar for children from low and high socio-economic deprivation households.

**Table 3:** Mean rate (95 %CI) of unhealthy food marketing exposure per day*

Exposure category	All participants (*n* 168)			Low household socio-economic deprivation (*n* 85)			High household socio-economic deprivation (*n* 77)		
	Rate per day*	95 % CI	% of total	Rate per day*	95 % CI	% of total	Rate per day*	95 % CI	% of total
Total	31·7	28·5, 35·3	100·0	30·9	26·9, 35·4	100·0	33·1	28·7, 38·3	100·0
Setting									
Home	9·0	8·0, 10·2	28·5	9·5	6·9, 13·2	30·8	8·1	5·1, 13·0	24·5
School	5·6	4·3, 7·2	17·5	5·3	3·7, 7·5	17·0	6·1	4·6, 8·2	18·4
Food venues†	4·1	2·7, 6·4	13·0	3·7	2·7, 5·0	11·9	4·8	1·9, 12·1	14·6
Recreation venues‡	2·2	1·1, 4·3	7·0	2·0	1·1, 3·4	6·3	2·6	1·1, 6·1	8·0
Other public spaces§	10·8	8·0, 14·5	34·0	10·5	8·0, 13·7	33·9	11·4	6·5, 20·1	34·5
Marketing medium									
Product packaging	17·6	15·9, 19·5	55·6	17·1	15·5, 18·9	55·5	18·4	13·9, 24·4	55·7
Sign	10·2	7·3, 14·2	32·0	9·5	7·3, 12·4	30·8	11·3	6·7, 19·2	34·1
In-store marketing	2·4	1·8, 3·2	7·6	2·3	1·6, 3·2	7·4	2·6	1·5, 4·6	8·0
Print media	0·7	0·2, 1·8	2·1	1·0	0·2, 4·0	3·2	0·2	0·1, 0·5	0·5
Screen	0·2	0·1, 0·4	0·6	0·2	0·1, 0·5	0·7	0·1	0·1, 0·2	0·4
Merchandise	0·7	0·3, 1·3	2·1	0·7	0·3, 1·8	2·4	0·4	0·1, 1·3	1·3
Product category									
Sugary drinks	9·1	8·2, 10·0	28·6	8·5	7·1, 10·3	27·6	9·9	7·4, 13·3	29·9
Fast food	5·9	4·6, 7·6	18·7	6·4	4·5, 9·2	20·9	5·1	3·5, 7·4	15·5
Confectionery	3·0	2·2, 4·0	9·4	2·5	1·8, 3·5	8·0	3·8	2·2, 6·5	11·5
Snack foods	2·8	2·3, 3·4	8·9	2·8	2·1, 3·7	9·2	2·8	2·4, 3·3	8·4
Ice cream	1·9	1·3, 2·7	5·9	1·7	1·1, 2·5	5·3	2·3	1·4, 3·6	6·8
Diet soft drinks	1·3	0·9, 1·9	4·2	1·7	1·2, 2·6	5·6	0·7	0·3, 1·7	2·1
Cookies/cakes/pastries	1·7	1·0, 2·8	5·4	1·5	1·1, 2·2	4·9	2·0	1·0, 4·0	6·0
Milk product (unhealthy)	0·8	0·4, 1·3	2·4	0·6	0·4, 0·9	1·9	1·0	0·5, 1·9	2·9
Cereal (unhealthy)	0·7	0·4, 1·1	2·1	0·8	0·4, 1·6	2·5	0·5	0·3, 0·8	1·6
Other	4·6	3·7, 5·7	14·5	4·4	3·3, 5·8	14·1	5·0	4·1, 6·3	15·2

* A day is defined as 10 h’ worth of images.

† Includes bakeries, convenience stores, fast food outlets, fresh food markets, full service restaurants and supermarkets.

‡ Includes community venues, outdoor recreations space and sports facilities.

§ Includes ‘no setting’, other retail, private transport, public transport, shop fronts, shopping malls, streets and vending machines.

### Comparison of policies to reduce unhealthy food marketing exposures

Table 4 shows the estimated food marketing exposures avoided per day and the percentage reduction in overall exposure for the ten scenarios. In order of effectiveness, the exposure reductions were 60·3 % for plain packaging, 28·8 % for no marketing of sugary drinks (32·7 % if including artificially sweetened beverages), 22·2 % for no marketing in public spaces (excluding product packaging), 19·2 % for no marketing in public spaces within 400 m of bus stops (excluding product packaging), 12·6 % for no marketing in public spaces within 400 m of schools (excluding product packaging), 8·2 % for no sugary drinks marketing in schools and 5·0 % for no marketing in public spaces within 400 m of main roads (excluding product packaging). Bans on merchandise marketing, confectionary in schools and marketing within 400 m of recreational facilities had minimal effect (< 5 % reduction). For bans around key settings (scenarios 7–10), effectiveness depended on the size of the ban area. For example, banning 400 m around bus stops (ban area = 8·96 % of the Wellington region) had a much greater effect than banning 400 m around recreational facilities (ban area = 1·11 % of the Wellington region).

**Table 4:** The estimated reduction in children’s exposure to unhealthy food marketing under different scenarios

Number	Scenario	Average daily food marketing exposures avoided*	95 % CI	Percentage reduction in food marketing exposure	95 % CI
1	No marketing on product packaging	17·6	15·9, 19·5	60·3	54·2, 67·2
2	No marketing of sugary drinks	9·1	8·2, 10·0	28·8	25·0, 33·0
3	No marketing on merchandise	0·7	0·3, 1·3	1·9	1·3, 2·8
4	No marketing of confectionary in schools	0·3	0·1, 0·8	1·7	0·7, 4·1
5	No marketing of sugary drinks in school	2·1	1·5, 3·0	8·2	6·1, 11·1
6	No outdoor marketing in public spaces†	8·4	5·6, 12·6	22·2	16·1, 30·6
7	No outdoor marketing within 400 m of schools†	4·8	3·2, 7·2	12·6	9·1, 17·4
8	No outdoor marketing within 400 m of recreation venues†	1·2	0·8, 1·8	3·1	2·3, 4·3
9	No outdoor marketing within 400 m of bus stops†	7·3	4·8, 10·9	19·2	13·9, 26·5
10	No outdoor marketing within 400 m of main roads†	1·9	1·3, 2·8	5·0	3·6, 6·9

* A day is defined as 10 h’ worth of images.

† Excludes product packaging.

### Comparison of policies to reduce unhealthy food marketing exposures outside home

As exposure to unhealthy food/beverages inside homes is outside the scope of many policies aiming to restrict marketing (e.g. those focused on outdoor signage), we performed a sub-analysis of key policies that excluded food marketing in the home setting (see Table 5). On average, children were exposed to unhealthy food marketing at home 9·0 times per day (28·5 % of total exposures); the vast majority of this exposure was on product packaging (59·4 % *v*. 55·6 % for all settings). After omitting home exposures, plain packaging remained the most effective policy scenario, although with a smaller percentage reduction (49·8 % *v*. 60·3 % for all settings). The percentage reduction for policies targeting public spaces (scenarios 4, 7, 8, 9 and 10) and schools (scenarios 5 and 6) increased after omitting home exposures. For example, a ban on marketing in all public spaces would reduce children’s exposure outside home by 30·4 %, compared with 22·2 % for all settings.

**Table 5:** Percentage reduction in children’s exposure to unhealthy food marketing under different scenarios, for all settings and non-home settings

Number	Scenario	All settings	95 % CI	All settings, omitting home	95 % CI
1	No marketing on product packaging	60·3	54·2, 67·2	49·8	42·9, 57·8
2	No marketing of sugary drinks	28·8	25·0, 33·0	26·7	23·0, 31·0
3	No marketing on merchandise	1·9	1·3, 2·8	1·4	0·7, 2·6
4	No marketing of confectionary in schools	1·7	0·7, 4·1	2·3	0·9, 5·7
5	No marketing of sugary drinks in schools	8·2	6·1, 11·1	12·0	9·1, 15·9
6	No outdoor marketing in public spaces*	22·2	16·1, 30·6	30·4	22·4, 41·2
7	No outdoor marketing within 400 m of schools*	12·6	9·1, 17·4	17·3	12·8, 23·4
8	No outdoor marketing within 400 m of recreation venues*	3·1	2·3, 4·3	4·3	3·2, 5·8
9	No outdoor marketing within 400 m of bus stops*	19·2	13·9, 26·5	26·3	19·4, 35·7
10	No outdoor marketing within 400 m of main roads*	5·0	3·6, 6·9	6·8	5·0, 9·2

* Excludes product packaging.

### Differences by socio-economic deprivation

**Fig. 1:**
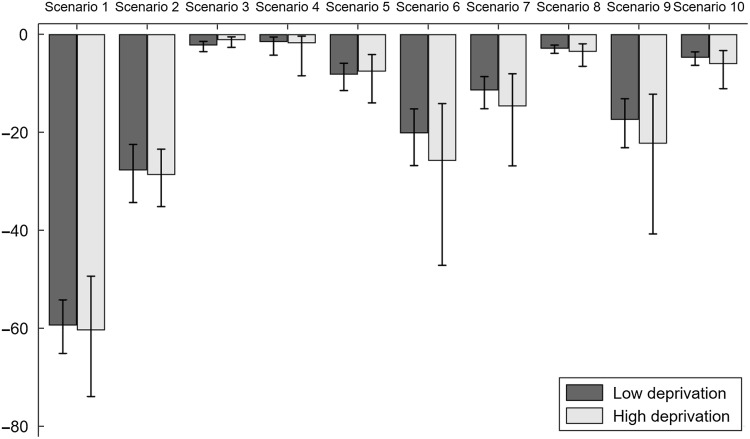
Percentage reduction (95 % CI) in children’s exposure to unhealthy food marketing under different scenarios by household socio-economic deprivation. Policy bans: Scenario 1 = No marketing on product packaging. Scenario 2 = No marketing of sugary drinks. Scenario 3 = No marketing on merchandise. Scenario 4 = No marketing of confectionary in schools. Scenario 5 = No marketing of sugary drinks in schools. Scenario 6 = No outdoor marketing in public places. Scenario 7 = No outdoor marketing within 400 m of schools. Scenario 8 = No outdoor marketing within 400 m of recreational venues. Scenario 9 = No outdoor marketing within 400 m of bus stops. Scenario 10 = No outdoor marketing within 400 m of major roads

Results were similar for children from high and low socio-economic deprivation households (Fig. 1). For children from high socio-economic deprivation households, larger reductions were found for a ban on outdoor marketing in public spaces and a ban on outdoor marketing within 400 m of bus stops, although the differences were not statistically significant.

## Discussion

Children in this study were exposed to unhealthy food marketing 31·7 times per day; this varied by setting, marketing medium and product category. Marketing occurred most in public spaces (34 %) and at home (28·5 %) and through product packaging (55·6 %) and signage (32 %) on sugary drinks (28·6 %), fast food (18·7 %) and confectionary (9·4 %). There were no exposure differences by socio-economic deprivation, a finding inconsistent with a recent systematic review^(17)^ that found that children from low socio-economic backgrounds were disproportionately exposed to unhealthy food marketing. The most effective of ten policy options for eliminating children’s exposure to unhealthy food marketing were no marketing: on product packaging (60·3 % reduction), of sugary drinks (28·8 %), in public spaces (22·2 %), in public spaces within 400 m of bus stops (19·2 %) in public spaces within 400 m of schools (12·6 %) of sugary drinks in schools (8·2 %) and in public spaces within 400 m of main roads (5·0 %). There was no significant difference in policy impacts by socio-economic deprivation.

### Strengths and limitations

This study incorporates the objective measurement of food marketing using wearable cameras and GPS devices. The unhealthy food exposure captured by wearable cameras mitigated the bias caused by spatial exposure estimation methods based on mobility and GIS data^(25)^. This study has several advantages compared with previous analyses of food marketing restrictions done by our team^(18,22)^. Specifically, we tested more banning scenarios to reflect the many feasible options available to policy makers and did so across four cities in a metropolitan region. We also compared policy effectiveness by socio-economic deprivation.

Limitations include the image capture rate (every seven seconds) and coding of brands if 50 % or more of a brand name or logo were seen, which likely underestimated children’s exposure^(3,26)^, especially for marketing on screens. The sample, while providing objective data, is relatively small. Some GPS coordinates were missing due to noise and loss of signal, which may have biased the findings towards children with more outdoor GPS data^(22)^. The impact of substitution effects (replacing one form of marketing with another) was not calculated in the policy analysis due to a lack of evidence.

### Implications for policy and research

Plain packaging of food was by far the most effective policy (60·3 %). Food packaging is an important area of marketing, whereby bright colours and the usage of cartoons and characters on packaging attract attention and ultimately consumption^(5,15)^. While plain packing of food is uncommon^(5)^, plain packaging of tobacco has been implemented in over twenty-two jurisdictions^(27)^. Further, Chile has banned the use of cartoon characters or mascots in unhealthy food marketing, including on food packaging^(28)^. Banning marketing of sugary drinks, including product packaging, reduced children’s exposure to marketing by more than a quarter (28·8 %). Sugary drinks have been an increasing focus for policy intervention with the introduction of sugary drinks taxes in over 100 jurisdictions^(29)^. This suggests policy on a single food type is possible, as Latvia has done with energy drinks^(12)^. School marketing bans reduced children’s exposure by smaller amounts, 8·2 % for sugary drinks and 1·7 % for confectionary. New Zealand schools have the authority to implement these policies, in line with WHO advice^(20)^.

This research indicates that there is value in bans within 400 m of schools with 12·6 % reduction in exposure which would more than halve children’s exposure to outdoor marketing. However, the study also demonstrates that children are exposed to marketing across a range of public spaces. Bans of marketing in public spaces within 400 m of bus stops (19·2 %) was one of the most effective settings-based policies. While restrictions on marketing in and around public transport systems are receiving increasing attention (including adoption in London, UK)^(30)^, although policies on this scale may have a considerable implementation burden.

This research suggests that it would be even more effective to ban all unhealthy food marketing in public spaces (22·2 %). This is likely a more feasible (and potentially attractive) option for policy makers. This would simplify implementation, reduce running costs and reduce substitution of unhealthy food marketing in non-restricted areas. São Paulo and Grenoble have taken this further and banned all marketing in public spaces^(31,32)^. This would eliminate the potential substitution of unhealthy food marketing with other harmful commodity marketing, for example, alcohol and gambling. Such policies could deliver equitable outcomes for children by socio-economic status, critical given the higher burden of obesity among children of low socio-economic status^(2)^.

Further research is needed to evaluate the impact and equity of these policy options, with particular attention to the impact of substitution. Given the increase in online marketing since data for this study were collected, detailed analysis in this setting is required, using objective methods of data collection, for example.^(33)^

## Conclusions

In this study, children were exposed to unhealthy food marketing over thirty times per day. Much of this exposure was from product packaging encountered in at home and in public spaces. When looking at the percentage reduction in food marketing exposure from ten policy scenarios, the most effective were plain packaging, bans on marketing sugary drinks (including packaging) and bans on marketing in public spaces, with no differences by socio-economic deprivation. Children encountered unhealthy food marketing in multiple settings from multiple marketing mediums throughout their day, highlighting the need for comprehensive unhealthy food marketing bans. Given the challenge of rising and inequitable childhood obesity globally^(1)^, such interventions are urgently needed, in order to equitably protect children’s right to health^(34)^.
